# Exposure to Blue Light Reduces Melanopsin Expression in Intrinsically Photoreceptive Retinal Ganglion Cells and Damages the Inner Retina in Rats

**DOI:** 10.1167/iovs.63.1.26

**Published:** 2022-01-21

**Authors:** Natalia Ziółkowska, Małgorzata Chmielewska-Krzesińska, Alla Vyniarska, Waldemar Sienkiewicz

**Affiliations:** 1 Department of Histology and Embryology, Faculty of Veterinary Medicine, University of Warmia and Mazury in Olsztyn, Olsztyn, Poland; 2 Department of Pathophysiology, Forensic Veterinary and Administration, Faculty of Veterinary Medicine, University of Warmia and Mazury in Olsztyn, Olsztyn, Poland; 3 Department of Pharmacology and Toxicology, Faculty of Veterinary Medicine, Stepan Gzhytskyi National University of Veterinary and Biotechnologies, Lviv, Ukraine; 4 Department of Animal Anatomy, Faculty of Veterinary Medicine, University of Warmia and Mazury in Olsztyn, Olsztyn, Poland

**Keywords:** melanopsin, rat, blue light, intrinsically photoreceptive retinal ganglion cells, retinal damage

## Abstract

**Purpose:**

The purpose of this study was to investigative the effects of blue light on intrinsically photoreceptive retinal ganglion cells (ipRGCs).

**Methods:**

Brown Norway rats were used. Nine rats were continuously exposed to blue light (light emitting diodes [LEDs]: 463 nm; 1000 lx) for 2 days (acute exposure [AE]); 9 rats were exposed to 12 hours of blue light and 12 hours of darkness for 10 days (long-term exposure [LTE]); 6 control rats were exposed to 12 hours of white fluorescent light (1000 lx) and 12 hours of darkness for 10 days. Whole-mount retinas were immunolabelled with melanopsin antibodies; melanopsin-positive (MP) ipRGC somas and processes were counted and measured with Neuron J. To detect apoptosis, retinal cryo-sections were stained with terminal deoxynucleotidyl transferase dUTP nick-end labeling. Ultra-thin sections were visualized with transmission electron microscopy.

**Results:**

The number of MP ipRGC somas was significantly lower in retinas from AE and LTE rats than in those from control rats (*P* < 0.001 and = 0.002, respectively). The mean length of MP areas of processes was significantly lower in AE rats (*P* < 0.001). AE rats had severe retinal damage and massive apoptosis in the outer nuclear layer; their mitochondria were damaged in the axons and dendrites of the nerve fiber layer and the inner plexiform layer. Retinal ganglion cells (RGCs) in AE rats appeared to have reduced amounts of free ribosomes and rough endoplasmic reticulum.

**Conclusions:**

AE to blue light reduces melanopsin expression and damages RGCs, likely including ipRGCs. Changes in the axons and dendrites of RGCs suggest possible disruption of intraretinal and extraretinal signal transmission.

As the use of devices emitting blue-enriched light has increased, so have concerns about its negative effects on the retina.^1^^,^^2^ Excessive exposure to blue light can cause insomnia and Transient Smartphone Blindness,^3^ damage the retina, and threaten vision.^4^^–^^7^ In general, the retina is most sensitive to shorter-wavelength light, which causes damage that is primarily photochemical in nature.^8^ Blue light causes photoreceptor death and retinal gliosis, which leads to retinal degeneration.^4^^–^^7^^,^^9^^–^^14^

Blue light exposure may also affect intrinsically photoreceptive retinal ganglion cells (ipRGCs), as these cells contain melanopsin, a photopigment with peak absorbance at around 480 nm.^15^ The ipRGCs play roles in image-forming responses^16^^,^^17^ and in non–image-forming responses.^1^^,^^17^ In the rat retina, these neurons constitute about 1% to 3% of the RGC population and are most concentrated in the supero-temporal region.^18^^,^^19^ High-intensity white light exposure decreases the number of ipRGCs in the rat retina, by downregulating melanopsin expression,^20^ and changes melanopsin localization within these cells.^21^

However, even though it is known that blue light emitting diodes can be more damaging to the retina than white or green light,^4^^,^^5^^,^^13^^,^^14^ the effects of monochromatic blue-light exposure on rat ipRGCs have not been investigated. The ipRGCs could be particularly susceptible to blue light irradiation because their axons are unshielded by myelin and laden with mitochondria.^22^^,^^23^ Mitochondrial enzymes are affected by blue light, which can lead to generation of reactive oxygen species and affect axonal transport and the survival of ipRGCs.^24^^–^^26^ Moreover, the only studies involving long-term exposure to blue light have investigated its effects on photoreceptors,^9^^,^^27^ not ipRGCs. For this reason, more long-term studies on animal models are needed. Additionally, in studies on the effects of exposure to high-intensity white light on ipRGCs, only albino rats were used. However, light-induced retinal degeneration differs in pigmented and albino rats,^28^ as does regulation of melanopsin mRNA and protein expression.^28^^–^^30^ Thus, the response of ipRGCs to light exposure may also differ between these strains, but studies on pigmented rats are needed to provide a basis for comparison.

Therefore, our objective was to determine the effects of acute and long-term exposure to blue light (1000 lx) on the retinas of pigmented rats, with a particular focus on ipRGCs. To examine and count ipRGCs in whole-mount retinas, we used immunolabeling for melanopsin. For measuring the length of ipRGC processes, we used Neuron J. We supplemented these techniques with electron microscopy, and for labeling apoptotic cells we used terminal deoxynucleotidyl transferase dUTP nick-end labeling (TUNEL).

## Methods

### Animals and Experimental Design

Three-month-old male and female Brown Norway rats (Charles River Laboratories) were used. All rats were kept in 12 hours of white fluorescent light and 12 hours of darkness for the first 3 months of life. Fluorescent lamps provided white light (≤ 100 lx). Then, the acute exposure (AE) group (9 rats) was first kept in total darkness for 24 hours, and then continuously exposed to blue light for 48 hours. The long-term exposure (LTE) group (9 rats) was exposed to 12 hours of blue light and 12 hours of darkness for 10 days. The control group (6 rats) was kept in 12 hours of white light (1000 lx) and 12 hours of darkness for 10 days. To dilate the rats’ pupils in all 3 groups, 1% atropine drops were applied twice a day.

All rats were kept in transparent, plexiglass cages (Supplementary Fig. S1). Blue light was provided by clusters of light emitting diodes (LEDs; 463 ± 10 nm; 400 blue diodes per cage; 50 diodes provide 16,000 lumen/5 meters^2^). White and blue LED illumination was measured at the level of the rats’ eyes with a monochromatic light meter (Multi-Led TENMARS, Taiwan). Supplementary Figure S1 shows other details of the experimental system. All rats were euthanized at 08:00 AM in a CO_2_ chamber. All experimental procedures were performed according to Ukrainian animal welfare regulations and were approved by the Poltava National Agricultural Academy Ethical Commission.

### Immunostaining

Rat retina whole mounts were prepared as in Dreising et al.^31^ Briefly, the eyes were enucleated immediately after euthanasia. Next, the retina was gently dissected from the eye cup and fixed in 4% paraformaldehyde for 45 minutes, then washed in PBS. For immunocytochemistry, the tissues were incubated for 48 hours with an antibody against melanopsin (1:200 dilution, abcam19383) in 0.1 M phosphate buffer containing 2% BSA and 0.1% Tween. After three 15-minute rinses in 0.1 M phosphate buffer, the tissues were incubated with secondary antibody (1:2000 dilution, Alexa 568) in 0.1 M phosphate buffer for 3 hours. Retinas were washed with PBS, then mounted in gelvatol mounting medium containing DABCO (Sigma, Germany) and Fluoromount G.^32^

### Counting of ipRGC Somas and Determination of Melanopsin-Positive Neural Process Lengths

Melanopsin-positive ipRGC somas in entire whole-mounts were counted manually with Image J 1.51n software.^33^ To determine neural process lengths, four images from the temporal retina were randomly selected from each retina and apposed (1.280 mm^2^ composite image). The same was done with 4 images from the nasal retina, for a total area of 2.560 mm^2^. Melanopsin immunoreactive nerve fibers were traced manually with Neuron J^34^ for Image J, and the length of all the fibers in the 2.560 mm^2^ area was summed. The ipRGC somas numbers in this area were also counted manually with Image J.

### TUNEL Assay

For apoptosis detection, a TUNEL assay was performed using an In Situ Cell Death Detection Kit (Roche Diagnostics GmBH, Mannheim, Germany) according to the manufacturer's instructions. Eight cross-sections of each retina were examined (Supplementary Table S1). TUNEL positive nuclei were counted manually in a 1000 µm long area of each section. In cases where nuclei may have fragmented, each potential fragment was counted as an individual nucleus.

### Electron Microscopy

For electron microscopy, the dorsal half of retinas was collected within 3 minutes of heart stoppage (see Supplementary Table S1). Tissue pieces were fixed (2 hours, 4°C) in a mixture of 1% paraformaldehyde and 2.5% glutaraldehyde in 0.2 M phosphate buffer (pH 7.4). Samples were washed, postfixed in 2% osmium tetroxide (2 hours at room temperature), and embedded in Epon 812. Semi-thin sections were stained with toluidine blue and examined to identify regions for electron microscopy. Ultrathin sections were contrasted with uranyl acetate and lead citrate, then examined using a Tecnai 12 Spirit G2 BioTwin transmission electron microscope (TEM; FEI, USA). Five to seven cross-sections of each retina were examined by TEM.

### Statistical Analyses

Differences in ipRGC soma numbers and melanopsin-positive neural-process lengths were analyzed with Tukey's HSD (R 4.0.4^35^). Sensitivity analysis with trimmed means^36^ indicated that the results were minimally affected by potential deviations from normality and heteroscedasticity.

## Results

### Exposure to Blue Light Reduced the Number of Melanopsin Positive ipRGC Somas in Rat Retinas

**Figure 1.**
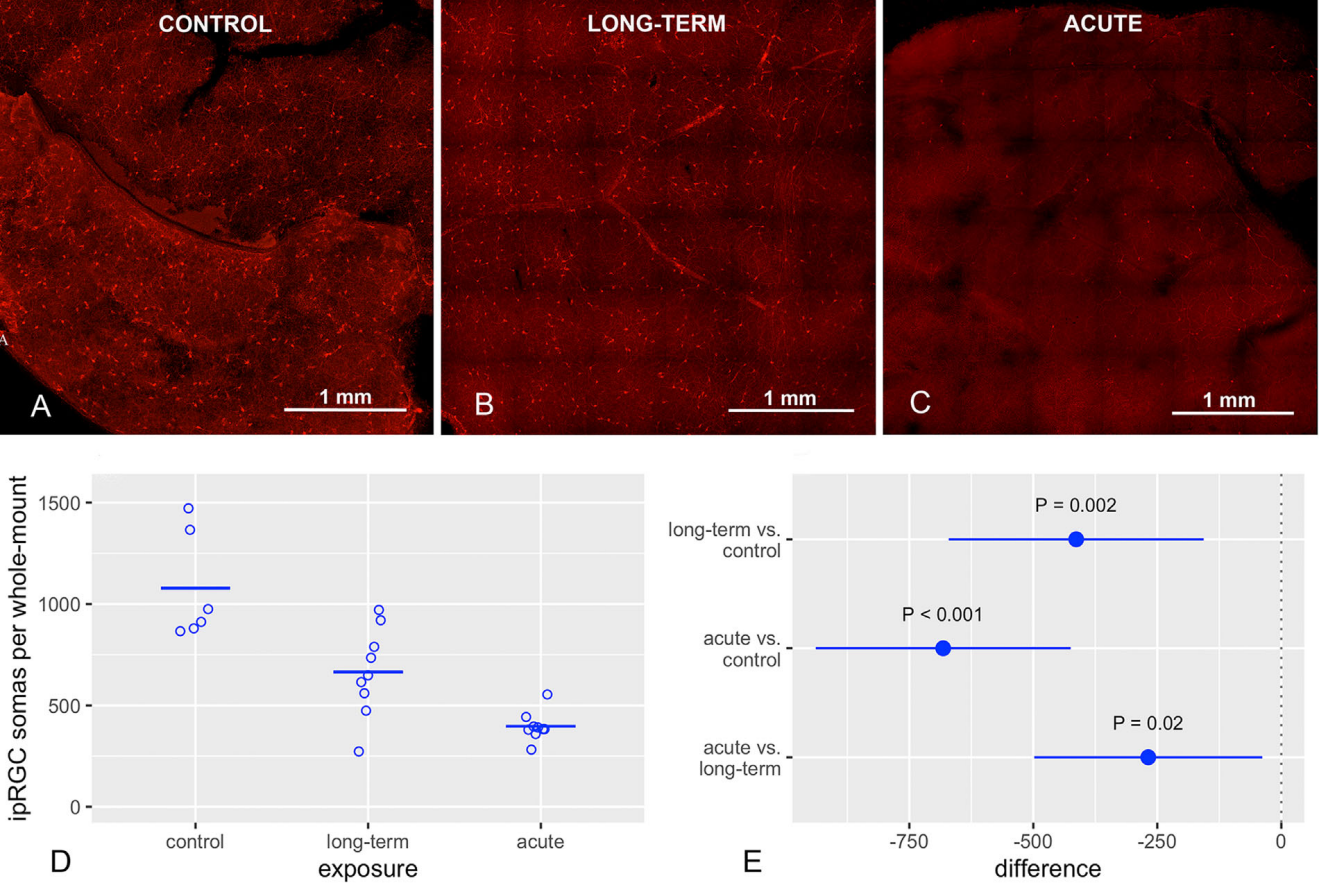
Blue light exposure reduced the number of intrinsically photoreceptive retinal ganglion cells (ipRGCs). Brown Norway rats were exposed to 12 hours of white light (1000 lx) and 12 hours of darkness for 10 days (control, 6 rats), 12 hours blue light (1000 lx), and 12 hours darkness for 10 days (long-term, 9 rats), and blue light (1000 lx) for 2 days (acute, 9 rats). Red fluorescence shows staining for melanopsin. (**A**) Control group, (**B**) long-term exposure group, and (**C**) acute-exposure group. (**D**) *Circles* indicate number of ipRGCs in one complete whole-mount retina from one rat; horizontal lines show mean. (**E**) *Dots* indicate differences between groups with regard to mean counts of ipRGCs in whole mount retinas; error bars show 95% confidence intervals for the differences. The *P* values and confidence intervals were calculated with Tukey's HSD test (R, version 4.0.4) after sensitivity analysis indicated that deviations from the assumptions of normality and homogenous variance would not affect conclusions about the statistical significance of differences.

**Figure 2.**
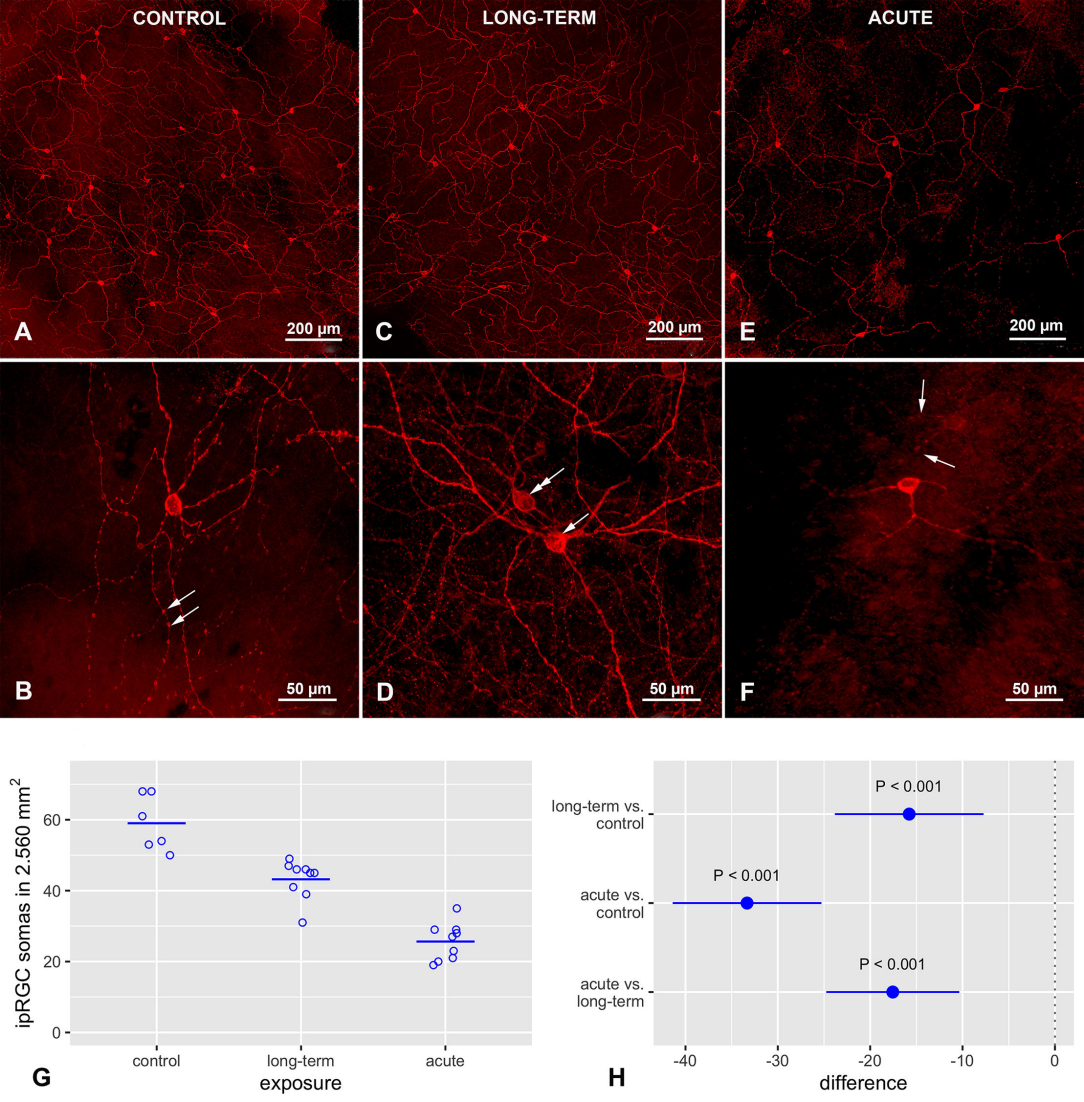
Effect of blue light exposure on melanopsin distribution in intrinsically photoreceptive retinal ganglion cells (ipRGCs) in whole mount retinas from Brown Norway rats. The rats were exposed to 12 hours of white light (1000 lx) and 12 hours of darkness for 10 days (control, 6 rats), 12 hours of blue light (1000 lx) and 12 hours of darkness for 10 days (long-term, 9 rats), and blue light (1000 lx) for 2 days (acute, 9 rats). (**A**) Control group (20×). Note: Dense network of melanopsin-positive dendrites. (**B**) Control group (40×). Multiple melanopsin-positive processes with varicosities are visible. (**C**) Long-term exposure group (20×). A wide-spread dendritic network of melanopsin-positive processes is visible. Note the smaller number of melanopsin-positive ipRGC somas. (**D**) Long-term exposure group (40×). Multiple melanopsin-positive varicosities are also visible here. Note: Two melanopsin-immunoreactive cells located in the ganglion cell layer (*arrow*) and in the inner nuclear layer (*double arrow*). (**E**) Acute exposure group (20×). Note: Levels of melanopsin expression differ widely in the ipRGC somas. Relative to the other groups, melanopsin immunoreactivity is decreased in the dendrites, and the melanopsin-positive varicosities on the nerve fibers are less prominent. (**F**) Acute exposure group (40×). Melanopsin is present mostly in ipRGC somas and proximal dendrites. Note: Lack of varicosities on these processes and decreased melanopsin immunoreactivity in outer stratifying dendrites (*arrows*). Samples were visualized with a confocal microscope (LSM700, Zeiss). (**G**) *Circles* indicate number of ipRGCs in a 2.560 mm^2^ area of one retina from one rat; horizontal lines show mean. (**E**) *Dots* indicate differences between groups with regard to mean counts of ipRGCs; error bars show 95% confidence intervals for the differences.

The AE and LTE groups had significantly fewer melanopsin positive ipRGC somas in the retina than the control group (Fig. 1). Although the AE group had significantly fewer of these somas in the retina than the LTE group, the 95% confidence interval (CI) for the difference included values near zero. Similar results were observed in the smaller 2.560 mm^2^ area that was used for measuring neural process lengths (see below) (Fig. 2): the AE group had significantly fewer melanopsin positive ipRGC somas than the LTE and control groups, and the LTE group had significantly fewer of these somas than the control group.

### Acute Exposure to Blue Light Changed Melanopsin Distribution in ipRGCs

Melanopsin distribution in ipRGCs was similar in the control and the LTE groups but differed in the AE group. The intensity of staining in the somas and processes was less intense in the AE group. In the AE group, immunoreactivity to melanopsin was sometimes limited to soma membranes. In most processes in the AE group, the staining did not extend as far from the soma as it did in the other groups (see Figs. 2A–F). In the AE group, melanopsin expression was reduced mostly in the outer stratifying dendrites of the inner plexiform layer (see Fig. 2F). Although melanopsin-positive varicosities were present on the processes in all three groups, they were less frequent and less prominent in the AE group (see Figs. 2E, 2F).

### Acute Exposure to Blue Light Reduced the Mean Length of ipRGC Neural Processes in Rat Retinas

**Figure 3.**
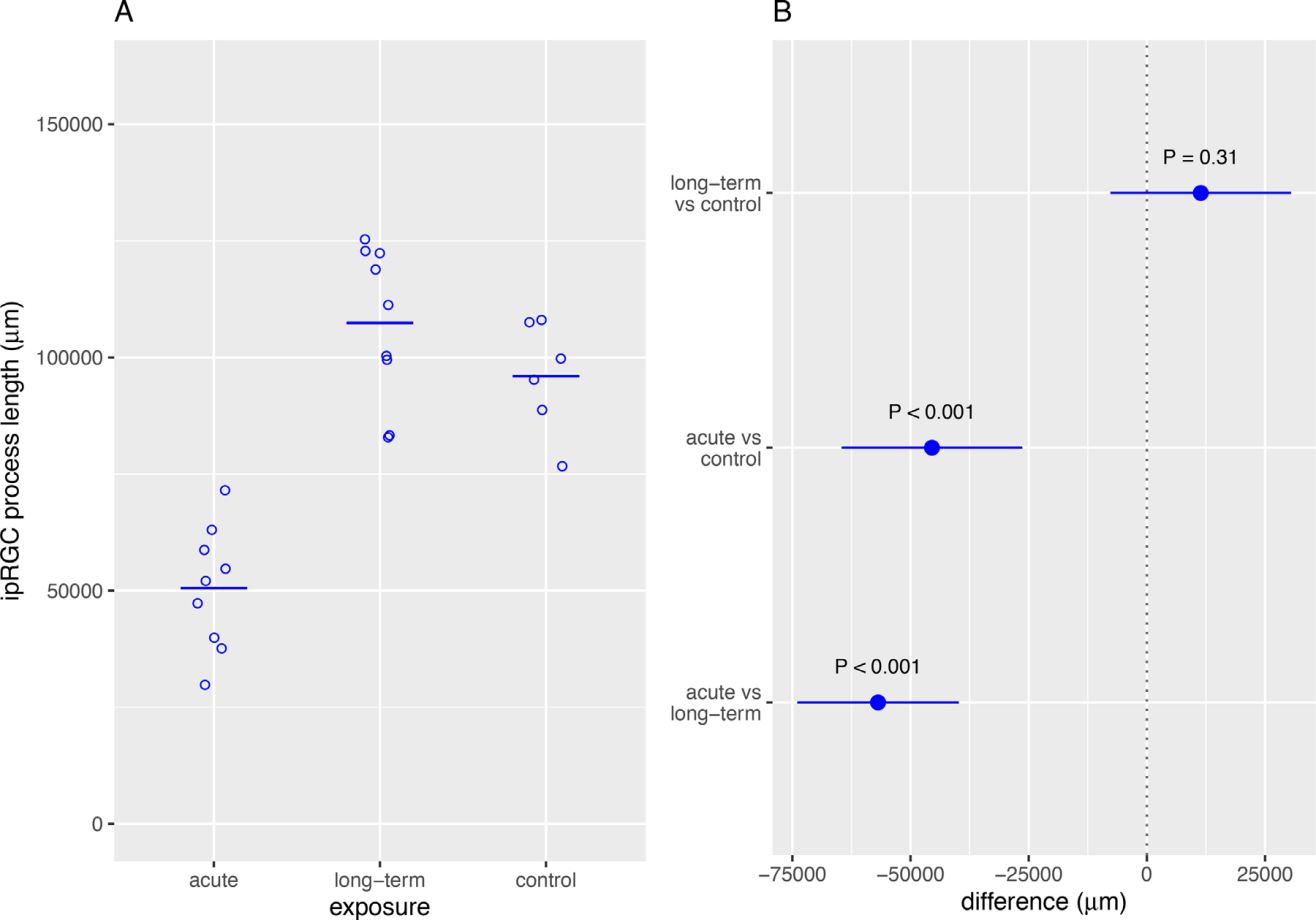
Acute exposure to blue light reduced the mean length of processes of intrinsically photoreceptive retinal ganglion cells (ipRGCs). Brown Norway rats were exposed to 12 hours of white light (1000 lx) and 12 hours of darkness for 10 days (control, 6 rats), 12 hours of blue light (1000 lx) and 12 hours of darkness for 10 days (long-term, 9 rats), and blue light (1000 lx) for 2 days (acute, 9 rats). (**A**) *Circles* indicate total length of ipRGC processes in a 2.560 mm^2^ area of the retina from one rat; horizontal line shows mean. (**B**) *Dots* indicate differences between groups, error bars show 95% confidence intervals for the differences.

The mean length of melanopsin-positive ipRGC neural processes in the examined 2.560 mm^2^ areas was significantly lower in the AE group than in the LTE and control groups (Fig. 3). The mean length of these nerve fibers was similar in the LTE and control groups.

### Retinal Damage After Blue-Light Exposure

**Figure 4.**
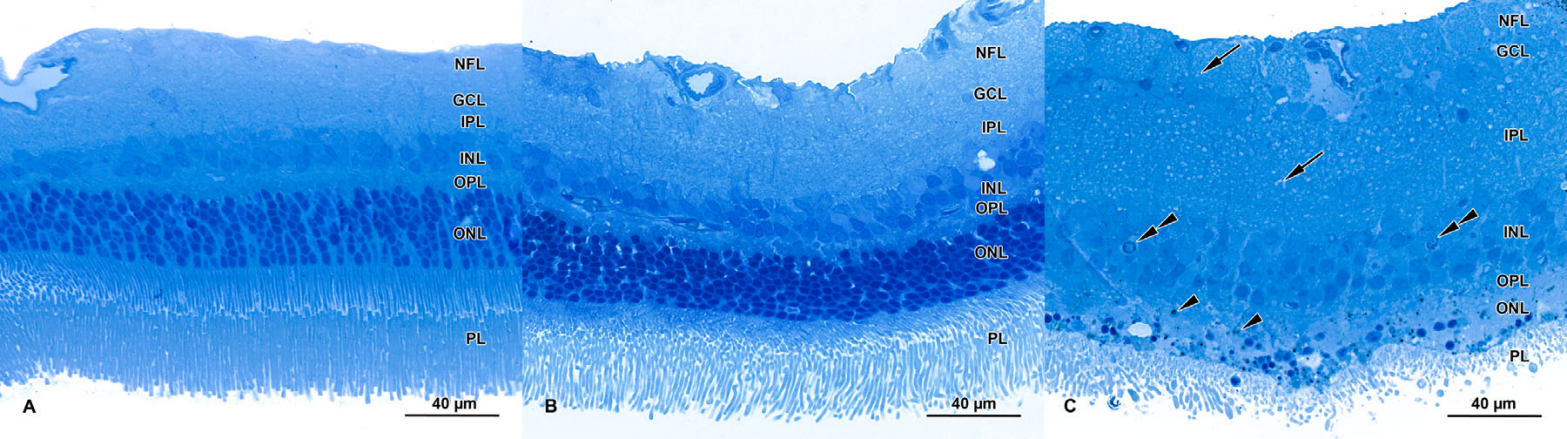
Effect of blue light exposure on retinal morphology of Brown Norway rats. The rats were exposed to 12 hours of white light (1000 lx) and 12 hours of darkness for 10 days (control, 6 rats), 12 hours of blue light (1000 lx) and 12 hours of darkness for 10 days (long-term, 9 rats), and blue light (1000 lx) for 2 days (acute, 9 rats). Resin-embedded semi-thin sections were stained with toluidine blue. Retinas from control (**A**) and long-term (**B**) exposure groups display normal morphology. (**C**) Retinas from the acute exposure group had deteriorated. Note: loss of nuclei in the outer nuclear layer. Photoreceptors are shrunken and sparser than in the other groups. Signs of apoptosis (chromatin condensation and marginalization) are present in the inner nuclear layer (*double arrow*). The inner plexiform layer and nerve fiber layer are thicker than in the control group and display vacuolization (*arrows*). Note the pigment granules scattered in the outer nuclear layer (*arrowheads*). Samples were visualized with a confocal microscope (LSM700, Zeiss).

Clear signs of retinal damage were visible in the AE group, some retinal abnormalities were detected in the LTE group, and no retinal abnormalities were seen in the control group. Light microscopy revealed that the photoreceptor layer had deteriorated in AE retinas (Fig. 4C) but not in LTE retinas (Fig. 4B). In AE retinas, the outer nuclear layer had disappeared, and only scattered nuclei were present (Fig. 4C). In those scattered nuclei, indicators of apoptosis were present (e.g. chromatin condensation; see Fig. 4C). Although the outer and inner photoreceptor segments were visible in most regions of AE samples, compared to the other groups, they were usually less numerous and shrunken (see Fig. 4C). In control and LTE samples, in contrast, the outer and inner segments had normal morphology. In the AE group, but not in the control and LTE groups (see Figs. 4A, 4B), the inner plexiform layer and the nerve fiber layer were thick and vacuolized (see Fig. 4C). In the inner nuclear layers (INLs) in the AE group, signs of apoptosis were occasionally present (e.g. chromatin condensation and marginalization; see Fig. 4C).

**Figure 5.**
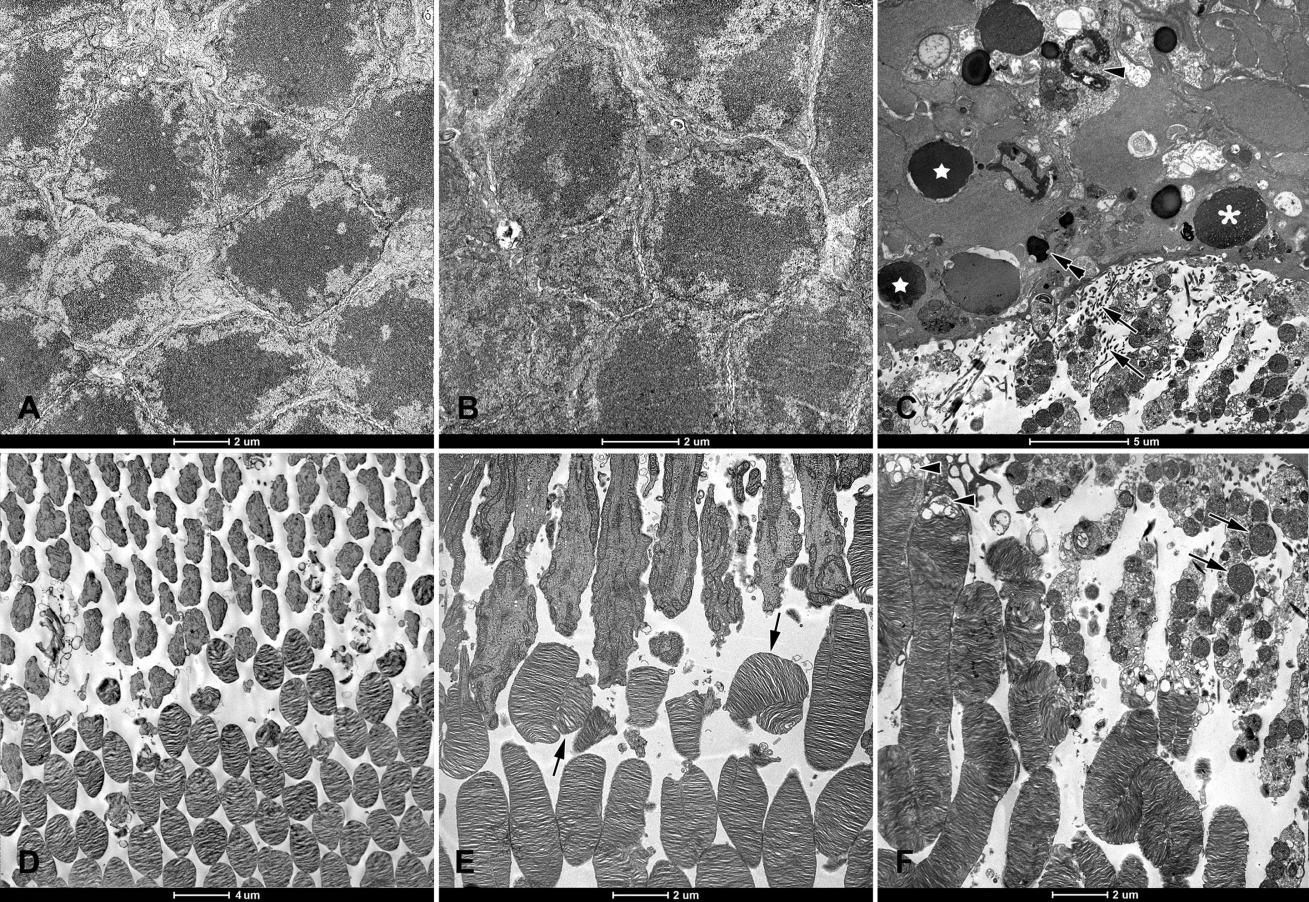
Electron micrographs of retinas from Brown Norway rats. The rats were exposed to 12 hours of white light (1000 lx) and 12 hours of darkness for 10 days (control, 6 rats), 12 hours of blue light (1000 lx) and 12 hours of darkness for 10 days (long-term, 9 rats), and blue light (1000 lx) for 2 days (acute, 9 rats). Normal looking outer nuclear layers (ONL) in (**A**) control and (**B**) long term exposure groups. (**C**) ONL from acute exposure group. Note: Nuclei are sparser, stages of apoptosis are evident (*star* = pyknosis; *asterisk* = chromatin pyknosis and vacuolization; and *arrowhead* = collapsed nuclei), and multiple processes of Müller cells are present between inner segments (*arrows*). Pigment granules are present between scattered nuclei (*double arrow*). Normal looking photoreceptor outer and inner segments from (**D**) control and (**E**) long-term exposure groups. Note: Some outer segments are disarranged (*arrows*). (**F**) Inner segments from acute exposure group have disrupted membranes and swollen mitochondria (*arrows*). Note: Vacuolization of the transition zone between photoreceptor inner and outer segments (*arrowheads*). Ultrathin sections were visualized using a Tecnai 12 Spirit G2 BioTwin transmission electron microscope (FEI, USA) equipped with two digital cameras: a Veleta (Olympus, Japan) and an Eage 4k (FEI, USA).

In electron micrographs, the outer nuclear layer looked normal in the LTE and control groups (Figs. 5A, 5B). In the AE group, however, there were markedly fewer nuclei in this layer, and cells displayed signs of different stages of apoptosis: pyknotic nuclei containing mostly heterochromatin, vacuolized nuclei, and nuclei that had collapsed (see Fig. 5C).

**Figure 6.**
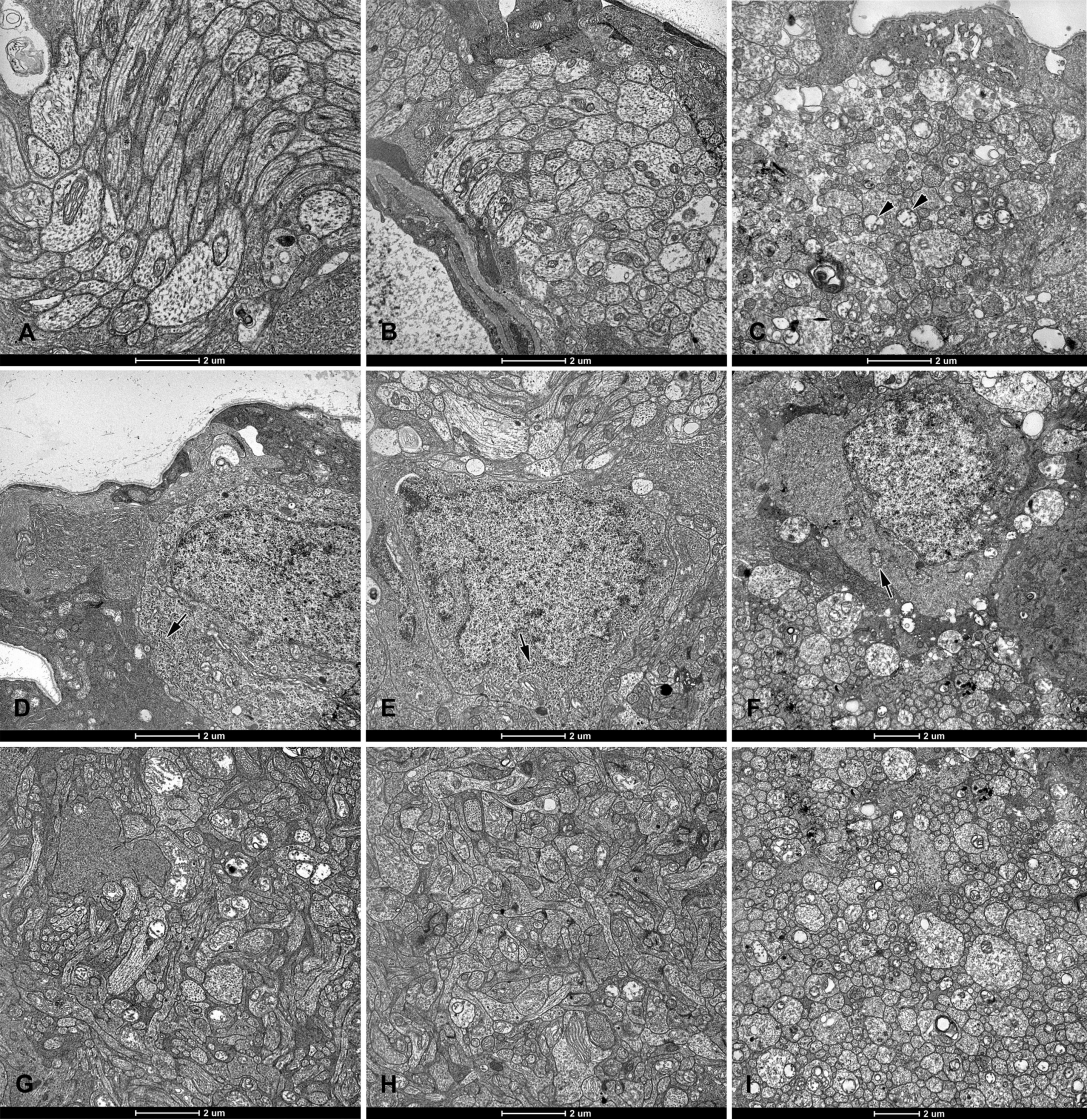
Electron micrographs of retinas from Brown Norway rats. The rats were exposed to 12 hours of white light (1000 lx) and 12 hours of darkness for 10 days (control, 6 rats), 12 hours of blue light (1000 lx) and 12 hours of darkness for 10 days (long-term, 9 rats), and blue light (1000 lx) for 2 days (acute, 9 rats). In the (**A**) control and (**B**) long-term exposure groups, nerve-fiber-layer axons and their mitochondria appear normal. (**C**) In the acute exposure group, axons are swollen and have mitochondria with disrupted cristae (*arrowheads*). Somas of ganglion cells from the (**D**) control and (**E**) long-term exposure groups display normal ultrastructure with numerous free ribosomes and cisterns of rough endoplasmic reticulum (*arrows*). (**F**) Ganglion cell somas from the acute exposure group have fewer free ribosomes and cisterns of the rough endoplasmic reticulum, as well as swollen mitochondria (*arrow*). In retinas from the (**G**) control and (**H**) long-term exposure groups, the inner plexiform layer appears normal. (**I**) In the retina from the acute exposure group, the inner plexiform layer is vacuolized. Ultrathin sections were visualized using a Tecnai 12 Spirit G2 BioTwin transmission electron microscope (FEI, USA) equipped with two digital cameras: a Veleta (Olympus, Japan) and an Eage 4k (FEI, USA).

Most of the inner segments looked normal in the LTE and control groups (see Figs. 5D, 5E). In the AE group, most inner segments had disrupted cell membranes and swollen mitochondria (see Fig. 5F). Photoreceptor outer segments were commonly disarranged in the AE group, and sometimes disarranged in the other two groups. In AE retinas, most of the rod outer segments had disarranged discs, and vacuoles were present at the transition zone between the inner and outer photoreceptor segments (see Figs. 5C, 5F). Multiple Müller cell processes were present in the subretinal space of AE rats, but not in retinas from the other rats. In AE retinas, these processes extended between inner photoreceptor segments (see Fig. 5C). In the AE group, the nerve fiber layer and the inner plexiform layer showed signs of damage (Figs. 6C, 6I): they were vacuolized, and the mitochondria in axons and dendrites were markedly swollen and had disrupted cristae. However, in the LTE and control groups, these layers looked normal (see Figs. 6A, 6B, 6G, 6H). Finally, ganglion cells appeared normal in control and LTE rats (see Figs. 6D, 6E). In AE rats, these cells had swollen mitochondria, and sparser ribosomes and cisterns of the rough endoplasmic reticulum (see Fig. 6F).

### TUNEL Assay for Apoptosis

**Figure 7.**
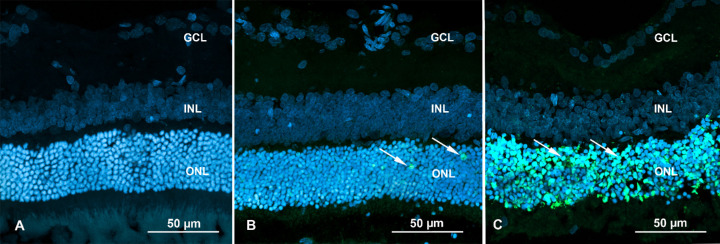
TUNEL assay of Brown Norway rat retinas. Blue fluorescence shows nuclei stained with DAPI. The rats were exposed to 12 hours of white light (1000 lx) and 12 hours of darkness for 10 days (control, 6 rats), 12 hours of blue light (1000 lx) and 12 hours of darkness for 10 days (long-term, 9 rats), and blue light (1000 lx) for 2 days (acute, 9 rats). (**A**) Retina from a rat in the control group. Note: No labeling for apoptosis. (**B**) Retina from a rat in the long-term exposure group. Isolated nuclei display labeling for apoptosis (*white arrows =* green fluorescence) in the ONL. (**C**) Retina from a rat in the acute exposure group. Note: Massive labeling for apoptosis is present in the ONL (*white arrows* = green fluorescence). Samples were visualized with a confocal microscope (LSM700, Zeiss). Abbreviations: ONL, outer nuclear layer; INL, inner nuclear layer; GCL, ganglion cell layer.

In control retinas, only isolated apoptosis signals were detected (2–6 nuclei per rat; Fig. 7, Supplementary Table S2). In LTE retinas, scattered individual nuclei were labeled positive in the ONL (12–27 nuclei per rat). In AE retinas, however, massive labeling was detected in the ONL, indicating widespread apoptosis of photoreceptors (approximately 3878–5126 nuclei per rat; note that nuclear fragmentation made exact quantification difficult). In AE retinas, occasional positive labeling was detected in individual nuclei in the INL.

## Discussion

Our results indicate that LTE and AE to blue light reduce the number of melanopsin-positive ipRGC somas in the retinas of pigmented rats. AE, but not LTE, substantially reduces the length of melanopsin-positive ipRGC neural processes and damages photoreceptors. Whereas LTE does not appear to affect retinal ganglion cells (RGCs), AE causes lesions in the axons in the nerve fiber layer and in the dendrites in the inner plexiform layer, as well as minor changes in RGC somas.

It is possible that ipRGCs may not have been detected because exposure to blue light could have reduced melanopsin expression in these cells. Thus, although we cannot be certain whether exposure to blue light reduces the number of ipRGCs, or whether it downregulates melanopsin expression in these cells, our results clearly indicate that ipRGCs are affected by exposure to blue light. In future studies, a marker for all RGCs will be used to clarify the effect of blue light on the entire population of these cells, and to elucidate differences in the response ipRGCs and other RGCs to blue-light exposure.

Our count of 900 to 1500 ipRGCs in retina whole mounts from normal pigmented rats is consistent with the results of other studies.^18^^,^^19^^,^^37^ Additionally, our observations that, in the LTE and AE groups, the number of ipRGC somas was reduced and that labeling for apoptosis was absent in the ganglion cell layer are similar to the findings of others,^20^^,^^21^ who exposed albino rats to white light (3000 lx for 2 days or 200 lx for 2–8 days). Light and darkness regulate melanopsin expression in the retinas of pigmented^28^ and albino rats,^38^ hamsters,^39^ and mice.^40^ Additionally, extended exposure of rats to constant white light for 1 to 5 days downregulates melanopsin expression, resulting in a reduced number of ipRGC somas.^38^ Taken together, these studies and ours indicate that the reduction in the number of ipRGC somas was not caused by cell death, but it could be due to reduced detection of somas caused by decreased melanopsin protein expression.

We found that AE to blue light reduces the total length of the melanopsin-positive areas of processes. There are at least three factors that could explain this finding and the reduction in the density of melanopsin-positive varicosities on those processes. First, downregulation of melanopsin expression may have played a role. Hannibal et al.^28^ showed that melanopsin expression was significantly reduced after a prolonged period of constant light exposure. Moreover, melanopsin protein expression is transiently downregulated in the retina of albino rats exposed to high-intensity cool white light*.*^20^ This downregulation is probably a protective mechanism because melanopsin produces reactive oxygen species when it is activated by blue light.^41^^–^^43^

Second, these effects of AE to blue light on ipRGCs could be due to changes in the distribution of melanopsin, which has been shown to change in rat retinas in response to constant illumination or constant darkness.^21^^,^^28^^,^^38^ For example, after 8 days of exposure to 200 lx of white light, melanopsin immunoreactivity was no longer detected in ipRGC processes and was seen only in their somas.^21^ Similarly, after 2 days in constant white light at 300 lx, melanopsin immunoreactivity was decreased in the distal dendritic processes; after 3 days, it was also decreased in the proximal processes; and after 5 days, it was only visible in the somas.^38^ Similar changes occurred in pigmented rats continuously exposed to white light, and the changes were more prominent in the outer stratifying processes than in the inner ones,^28^ as we also observed in our rats after continuous exposure to monochromatic blue light. Hannibal et al.^28^ postulated that newly synthesized melanopsin is transported from the soma to the processes and that the soma membrane may be the last place where melanopsin expression is downregulated in response to light exposure. In our rats, the mean length of melanopsin-positive processes in an area of 2.56 mm^2^ was slightly longer in the LTE group than in the control group. However, the 95% CI indicates that, in all such rats, the mean length of these areas could be reduced after LTE to blue light. Thus, our data indicate that AE reduces the mean length of melanopsin positive areas in ipRGC processes and leaves open the possibility that LTE could also reduce their length, but to a lesser extent.

Third, these effects on ipRGCs might also be due to photoreceptor damage. However, it remains unclear whether photoreceptors play a role in regulating melanopsin expression in ipRGCs. On the one hand, some studies indicate that classical photoreceptors interact with ipRGCs,^39^^,^^44^^–^^46^ and that ipRGCs form synapses with bipolar and amacrine cells, suggesting that rod and cone input may be involved in regulating melanopsin expression.^23^^,^^44^ In mice, the outer stratifying dendrites of ipRGCs are in close proximity to cone photoreceptor terminals, which suggests a functional connection.^45^ In transgenic mice with degenerated photoreceptors, melanopsin expression was undetectable.^30^ Additionally, Wan et al.^47^ found that, 28 days after rats were injected intraperitoneally with N-methyl-N-nitrosourea, their photoreceptors had degenerated and melanopsin protein expression in ipRGCs had decreased markedly, although it persisted in the soma for a long time. They suggested that intact photoreceptors may be necessary for the normal distribution of melanopsin in ipRGCs. On the other hand, some studies suggest that melanopsin expression by ipRGCs is independent of classical photoreceptor input. For example, the appearance of ipRGCs and melanopsin distribution within these cells did not differ between rodless/coneless mice and age-matched wild-type mice.^48^ In mice lacking certain enzymes that regulate the visual cycle, the melanopsin photocycle can occur independently of both the rod and cone photocycles.^49^ In neonatal albino rats, in which rods and cones are not fully developed, changes in melanopsin protein expression were independent of rod and cone input.^39^ Moreover, 1 month after albino rats were exposed to white light (3000 lx), melanopsin expression began to recover gradually while photoreceptors continued to die.^20^ Some of these discrepancies between studies may be due to differences in the experimental protocols and the models that were used (i.e. light-induced retinal damage versus chemically induced damage versus knockout rodent models). However, it remains unclear whether the damage to the photoreceptors in our rats was related to the changes in their ipRGCs.

Several of our observations suggest that AE to blue light damaged the ipRGCs. First, the melanopsin-positive varicosities were less numerous and less prominent in the AE group than in the other groups. Second, in the inner plexiform layer, all dendrites were vacuolized and had swollen mitochondria with disrupted cristae. Third, in the nerve fiber layer, the same lesions were observed in all the axons. Fourth, in RGCs in the ganglion cell layer, the number of free ribosomes and of cisterns of the rough endoplasmic reticulum appeared to be lower in the AE group than in the other groups. Apoptosis signals were not detected in the ganglion cell layer, but it is possible that, if the exposure had been prolonged, these cells would have undergone apoptosis. Although ipRGCs cannot be distinguished from other RGCs by electron microscopy, all RGCs in these sections displayed these abnormalities. To identify possible differences in the degree to which RGCs and ipRGCs respond to blue-light exposure, future studies could use immuno-electron microscopy.

These lesions and changes in the ipRGCs may be due to several causes. RGCs are susceptible to blue-light exposure because their axons and dendrites contain numerous mitochondria.^22^^,^^23^ These organelles can be harmed by short-wavelength blue light, which causes oxidative stress and reduces RGC cell survival.^50^^,^^51^ The varicosities in ipRGCs contain large numbers of mitochondria,^23^ which suggests that the mitochondrial lesions we observed could have been related to the reduced number and prominence of melanopsin-positive varicosities*.* The ipRGCs can be more resistant than other RGCs to some injuries like axotomy or optic nerve injury,^52^^–^^54^ but in other models (e.g. experimental glaucoma or inherited photoreceptor degeneration), the ipRGC death rate is similar to that of rods and cones.^55^^,^^56^ The ipRGCs seem to be more resistant to direct damage from light exposure than conventional photoreceptors.^4^^,^^6^^,^^9^ However, our results indicate that AE to blue light was probably sufficient to damage ipRGCs, and that this damage was probably the direct result of blue-light exposure, as there was not enough time for remodeling processes to damage these cells.^26^^,^^57^

The molecular mechanism of light-induced damage to RGCs and ipRGCs remains unclear.^58^ Although melanopsin is downregulated after light exposure, it could have contributed to ipRGC damage. As mentioned above, when melanopsin is activated by blue light, it produces reactive oxygen species.^41^^–^^43^ Light-activated melanopsin may open transient photoreceptor potential calcium-ion channels in the cell membrane, leading to an influx of calcium ions and further production of reactive oxygen species in the mitochondria.^58^ Investigating the potential role of melanopsin in direct damage to ipRGCs from light exposure would be an interesting topic for future studies.

We speculate that the lesions observed in the RGCs, which could include ipRGCs, may have had at least two effects. First, because the mitochondria in the axons and dendrites of these cells may help to meet the high energy demands of signal transmission,^24^ the mitochondrial lesions may have disrupted signal transmission between ipRGCs, other retinal neurons, and various brain regions responsible for image and non-image forming responses. Second, the changes in the rough endoplasmic reticulum, coupled with the reduction in the number of free ribosomes, may explain the reduction in melanopsin staining that we saw, which is consistent with quantitative studies on the effects of light exposure on melanopsin expression.^20^ Because different types of ipRGCs are involved in image-forming and non-image-forming functions,^59^^,^^60^ it would be interesting to investigate whether all types of ipRGCs are equally vulnerable to blue light.

In summary, we found that exposure to blue light reduces melanopsin immunoreactivity in the ipRGCs of pigmented rats. We showed that, although continuous exposure to blue light at 1000 lx for 2 days does not cause RGC death, it damages the mitochondria in axons and dendrites of RGCs, likely including ipRGCs. This suggests possible disruption of signal transmission between ipRGCs and other retinal neurons, and between ipRGCs and different regions of the brain responsible for image and non-image forming responses. Future work could use immuno-electron microscopy to study ipRGC morphology after blue light exposure and investigate whether and to what extent melanopsin is involved in direct damage to ipRGCs from light exposure.

## Supplementary Material

Supplement 1

Supplement 2

Supplement 3
