# Supplementary figures and images for: Exposure to Blue Light Reduces Melanopsin Expression in Intrinsically Photoreceptive Retinal Ganglion Cells and Damages the Inner Retina in Rats
Source: Invest Ophthalmol Vis Sci. 2022 Jan 21;63(1):26. doi: 10.1167/iovs.63.1.26 (PMC8787613; doi:10.1167/iovs.63.1.26)

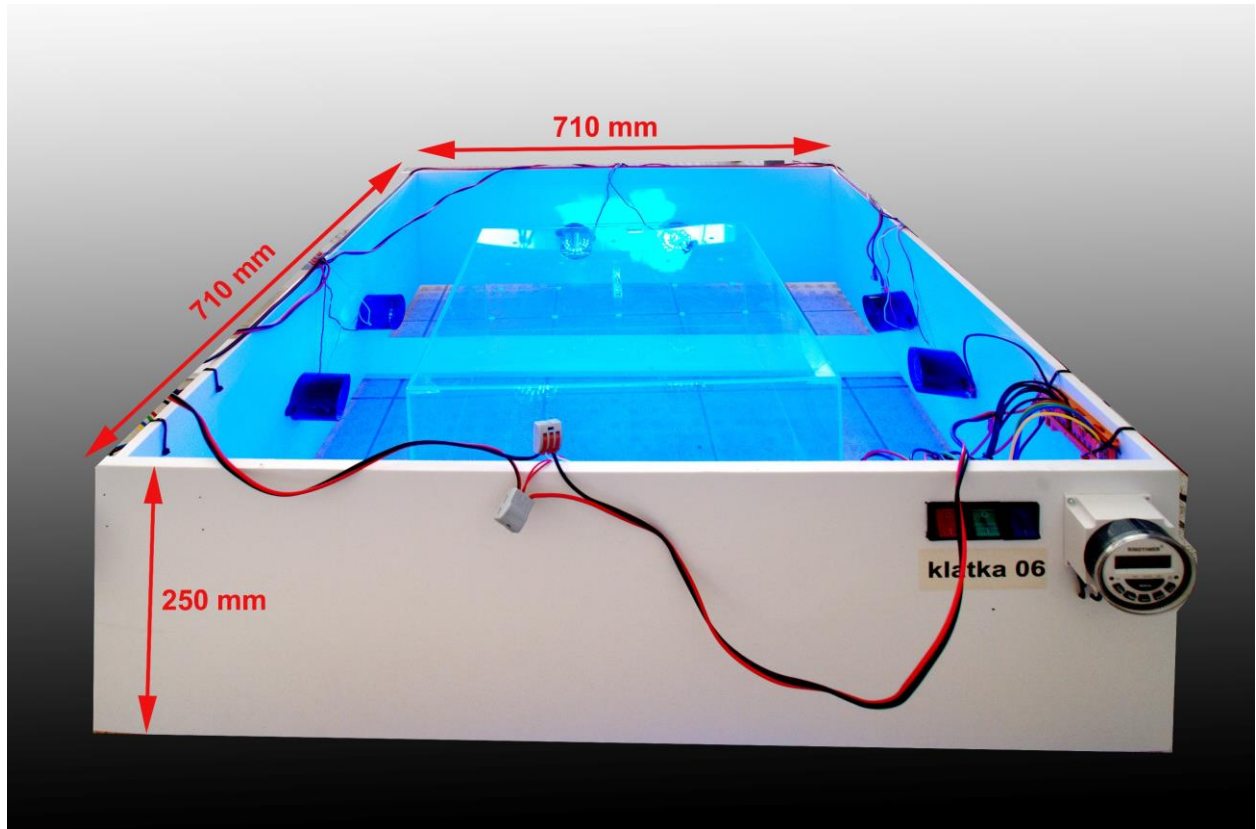

**Supplementary Figure 1.** Enclosure for experimental light exposure.

Supplement: Supplement 1 [file iovs-63-1-26_s001.pdf]
