# Supplementary material for: Exposure to Blue Light Reduces Melanopsin Expression in Intrinsically Photoreceptive Retinal Ganglion Cells and Damages the Inner Retina in Rats
Source: Invest Ophthalmol Vis Sci. 2022 Jan 21;63(1):26. doi: 10.1167/iovs.63.1.26 (PMC8787613; doi:10.1167/iovs.63.1.26)
Supplement: Supplement 2 [file iovs-63-1-26_s002.pdf]

**Animal number**	**Whole mount immunocytochemistry** [a]						**TUNEL** [b]						**Transmission electron microscopy** [c]					
	**Acute**		**Long term**		**Control**		**Acute**		**Long term**		**Control**		**Acute**		**Long term**		**Control**	
	**Right**	**Left**	**Right**	**Left**	**Right**	**Left**	**Right**	**Left**	**Right**	**Left**	**Right**	**Left**	**Right**	**Left**	**Right**	**Left**	**Right**	**Left**
Rat 1 ♀	+		+		+			+		+		+		+		+		+
Rat 2 ♂		+		+		+	+		+		+		+		+		+	
Rat 3♀	+		+		+			+		+		+		+		+		+
Rat 4 ♂		+		+		+	+		+		+		+		+		+	
Rat 5♀	+		+		+			+		+		+		+		+		+
Rat 6 ♂		+		+		+	+		+		+		+		+		+	
Rat 7♀	+		+					+		+				+		+		
Rat 8 ♂		+		+			+		+				+		+			
Rat 9 ♀	+		+					+		+				+		+		

Supplementary Table 1. Assignation of retinas for assays

[a] For whole mount immunocytochemistry, the entire retina from one eye was used; [b] For terminal deoxynucleotidyl transferase dUTP nick-end labelling (TUNEL), the nasal half of the eye was used; [c] For transmission electron microscopy, the temporal half of the eye was used
