# Supplementary material for: Exposure to Blue Light Reduces Melanopsin Expression in Intrinsically Photoreceptive Retinal Ganglion Cells and Damages the Inner Retina in Rats
Source: Invest Ophthalmol Vis Sci. 2022 Jan 21;63(1):26. doi: 10.1167/iovs.63.1.26 (PMC8787613; doi:10.1167/iovs.63.1.26)
Supplement: Supplement 3 [file iovs-63-1-26_s003.pdf]

Supplementary Table 2. Counts of TUNEL- positive nuclei in the outer nuclear layer of control rats and of rats exposed to blue light

Section	Acute exposure									Long term exposure									Control exposure					
	Animal									Animal									Animal					
	1	2	3	4	5	6	7	8	9	1	2	3	4	5	6	7	8	9	1	2	3	4	5	6
1	427	718	584	605	584	596	633	586	566	4	2	5	3	1	3	1	2	4	0	0	0	0	0	0
2	603	674	577	606	621	543	652	634	594	4	4	3	2	1	2	3	2	3	0	0	0	0	1	0
3	463	520	592	678	537	678	715	570	565	3	2	4	3	3	4	0	0	1	0	1	0	0	0	0
4	332	690	606	521	550	578	713	671	636	0	3	5	2	1	4	0	1	0	0	1	1	1	1	1
5	430	647	592	648	672	682	627	683	701	3	3	0	4	4	3	0	1	5	1	1	0	1	1	0
6	578	668	689	535	574	526	525	608	647	3	4	3	4	2	4	0	1	0	1	0	1	1	0	0
7	462	660	567	663	612	673	590	686	543	1	5	1	0	2	3	5	3	2	0	0	0	1	0	0
8	583	549	636	612	525	712	662	555	580	2	4	4	2	5	4	3	2	4	0	1	1	2	0	1
**SUM**	**3878**	**5126**	**4843**	**4868**	**4675**	**4988**	**5117**	**4993**	**4832**	**20**	**27**	**25**	**20**	**19**	**27**	**12**	**12**	**19**	**2**	**4**	**3**	**6**	**3**	**2**
**MEAN**	**4813.33**									**20.11**									**3.33**					
**SD**	**379.30**									**5.62**									**1.51**					
